# Comparison of Existing and New Total Knee Arthroplasty Implant Systems From the Same Manufacturer: A Prospective, Multicenter Study

**DOI:** 10.5435/JAAOSGlobal-D-21-00136

**Published:** 2021-12-15

**Authors:** William G. Hamilton, Ivan J. Brenkel, Steven L. Barnett, Paul W. Allen, Kimberly A. Dwyer, James P. Lesko, Stephen R. Kantor, Mark G. Clatworthy

**Affiliations:** From the Anderson Orthopaedic Research Institute, Alexandria, VA (Dr. Hamilton); Victoria Hospital, Department of Orthopaedic Surgery, Kirkcaldy, Fife, Scotland (Mr. Brenkel); the Orthopaedic Specialty Institute, Irvine, CA (Dr. Barnett); The Princess Alexandra Hospital, Harlow, UK (Mr. Allen); DePuy Synthes Joint Reconstruction, Warsaw, IN (Dr. Dwyer and Dr. Lesko); New London Hospital, New London, NH (Dr. Kantor); and Auckland Bone & Joint Surgery, Auckland, NZ (Dr. Clatworthy).

## Abstract

This study evaluated total knee arthroplasty (TKA) outcomes for an Existing-TKA versus New-TKA from the same manufacturer.

**Methods:**

TKA outcomes for 752 with Existing-TKA versus 1129 subjects with New-TKA were followed through 2 years using patient-reported outcome measures (PROMs). Responders were assessed per Outcome Measures in Rheumatology-Osteoarthritis Research Society International criteria. Kaplan-Meier implant survivorship was estimated. Radiographs had an independent radiographic review.

**Results:**

Two-year follow-up was 84.6% (636/752) for Existing-TKA and 82.5% (931/1129) for New-TKA. Two-year PROMs mean outcomes for New-TKA versus Existing-TKA at 2 years were: Knee Injury and Osteoarthritis Outcome Score (ADL: 89.0 versus 86.8, *P* = 0.005; pain: 88.9 versus 87.1, *P* = 0.019; symptoms: 84.1 versus 82.2, *P* = 0.017; Sport/Rec: 63.9 versus 58.8, *P* = 0.001; and QOL: 77.0 versus 73.5, *P* = 0.003), Patient's Knee Implant Performance (overall: 76.5 versus 73.5, *P* = 0.003; confidence: 8.4 versus 8.1, *P* = 0.004; stability: 8.6 versus 8.3, *P* = 0.006; satisfaction: 8.3 versus 8.1, *P* = 0.042; and modifying activities: 6.6 versus 6.4, *P* = 0.334), Oxford Knee Score (41.9 versus 41.1, *P* = 0.027), and EQ5D-3L (0.88 versus 0.88, *P* = 0.737). Two-year responder rates using WOMAC were 93.9% versus 90.6% (*P* = 0.018) for New-TKA versus Existing-TKA. Independent radiographic review showed that tibial and femoral radiolucencies ≥2 mm were similar (*P* ≥ 0.05) or favored New-TKA. Implant survivorship was similar between groups (log-rank *P* = 0.9994).

**Discussion:**

New-TKA versus Existing-TKA demonstrated slightly better PROMs with similar radiographic and implant survivorship outcomes.

Total knee arthroplasty (TKA) has evolved into highly successful surgery, providing excellent implant survivorship,^1,2^ pain relief,^3^ and improved quality of life^4,5^ for most patients who elect to have the surgery. However, up to 30%^5,6,7,8,9,10^ of patients reported dissatisfaction with the outcome of their surgery. New-TKA implants and surgical processes are intended to improve performance, particularly from a patient perspective. Infrequently, there are accompanying data to document relative clinical performance of newly released implants. The goal of this study was to evaluate clinical, patient-reported, implant survivorship, adverse events, and radiographic outcomes of an implant widely used for decades (Existing-TKA), compared with a new implant (New-TKA) from the same manufacturer. As a matter of surveillance, we wanted to confirm that there was no evidence of worsened outcomes with the new implant and, at the same time, to determine whether we could detect any improvements in outcomes in the short term, as recommended by Callaghan.^11^

## Methods

### Study Design

This was a prospective, nonrandomized multicenter clinical study of two total knee arthroplasty (TKA) implant systems, an Existing-TKA (PFC SIGMA) and a New-TKA (ATTUNE), both from the same manufacturer (DePuy Synthes Joint Reconstruction). The New-TKA is similar to the Existing-TKA design with some design modifications, including expanded size range, modified J-curve with gradually reducing femoral radii (versus multiradius) designed to improve AP kinematics, narrower and thinner anterior flange, proportional intracondylar box that allows for less bone removal in smaller patients, finer increments of patellar thickness, an extended trochlear groove, and 1-mm increments in polyethylene inserts. Approval was granted from each participating center's institutional review board or ethics committee, and written informed consent was provided by all study subjects before their enrollment. To avoid potential bias, neutral language was used in the consent forms when describing standard-of-care implants in both cohorts.

This study was a post hoc comparison of two separate trials in the United States, United Kingdom, Australia, and New Zealand. From October 2011 to March 2015, 27 surgeons at 19 sites consecutively enrolled 752 subjects with Existing-TKA. Between November 2012 and May 2015, 32 surgeons at 23 sites (of which 19 surgeons at 18 sites had enrolled Existing-TKA) consecutively enrolled 1,129 subjects with New-TKA. Subjects from sites which participated in both studies comprised 625 of 752 (83.1%) of Existing-TKA enrollment and 789 of 1,129 (69.9%) of New-TKA enrollment. The mean, minimum (% of total), and maximum (% of total) enrollment across Existing-TKA sites was 39.6, 9 (1.2%), and 77 (10.2%), and across New-TKA sites, it was 47.0, 4 (0.4%), and 100 (8.9%). At a given site, enrollment was nonoverlapping: Each site completed their Existing-TKA enrollment before commencing enrollment of New-TKA. In both cohorts, “consecutive enrollment” meant that all subjects who met eligibility criteria, including consent, were enrolled. Eligibility was identical in both cohorts: age 22 to 80 years, noninflammatory degenerative joint disease, informed consent, willing/able to follow protocol-defined clinic visits; exclusion criteria were if pregnant or lactating, contralateral knee in this study, contralateral amputation, prior knee arthroplasty (unicompartmental or total) patellectomy or high tibial osteotomy in the study knee, bedridden, current radicular pain from spine, enrolled in IDE/IND clinical investigation within past 3 months, involved in personal injury litigation or medical-legal or worker's compensation claims, drug or alcohol abuser, fibromyalgia being treated with prescription medication, neurological or musculoskeletal disorders that may affect gait or weight bearing (eg, muscular dystrophy, multiple sclerosis, and Charcot disease), inflammatory arthritis, or less than 2 years of life expectancy. Subjects were not compensated for their participation. The first 10 New-TKA learning curve cases that each surgeon implanted were included in this study and have also been analyzed separately to explore the technically demanding skills associated with New-TKA adoption.^12^ Both cohorts included all four TKA configurations (cruciate-retaining fixed bearing, cruciate-retaining rotating platform, posterior-stabilized fixed bearing, and posterior-stabilized rotating platform). Study investigators were trained on the New-TKA before enrollment and most attended cadaveric-based training. Most of the investigators were assigned to implant one configuration, consistent with their standard of care, with several exceptions where surgeons also subsequently enrolled in an additional configuration to help the team complete enrollment. All surgeons followed their preferred surgical technique for anterior/posterior referencing, femur first versus tibia first, gap balancing versus measured resection, cement choice, cementing technique, and patella resurfacing.

### Data Collection

Subjects were evaluated preoperatively and postoperatively at regular intervals. The postoperative intervals were <1 year (1—303 days), 1 year (304—668 days), and 2 years (669—1763 days) after surgery. Data collection included a broad range of patient-reported outcome measures (PROMs): Knee Injury and Osteoarthritis Outcome Score (KOOS)^13,14^ (enables WOMAC scoring), Oxford Knee Score,^15^ Patient's Knee Implant Performance,^16,17^ and EQ5D-3L.^18^ The original American Knee Society Score (AKS)^19^ was collected for Existing-TKA, and the 2011 AKS^20,21^ was collected along with the original AKS pain score (to allow for original AKS scoring) for New-TKA; range of motion (ROM) defined as flexion minus extension was collected for both. Because of tibiofemoral alignment differences between original and 2011 AKS questionnaires, the alignment component of the AKS score was taken from the independent laboratory radiographic assessments for both Existing-TKA and New-TKA cohorts instead of the clinical examination alignment collected by investigators. The PROMs data were also analyzed using responder analysis. Responders were assessed at 2 years according to the Outcome Measures in Rheumatology-Osteoarthritis Research Society International (OMERACT-OARSI)^22,23^ using both WOMAC and KOOS outcomes, where high responders were those who demonstrated at least a 50% and 20-point improvement on either pain or function score, and moderate responders were those who demonstrated at least a 20% and 10-point improvement in 2 of 3 scores: pain, function, or quality of life.

Subjects were only counted once for each respective AE, even if they reported the AE more than once, and percentages of subjects who experienced local adverse events were compared. Radiographs were prospectively collected in the New-TKA cohort (1118 provided preoperative radiographs; 922 provided minimum 2-year radiographs). For the Existing-TKA cohort, a pragmatic sample approach was used; all sites were asked to retrospectively provide their standard-of-care radiographs, but not all sites were able. Each site that was able, submitted all available standard-of-care radiographs that included 497 provided preop radiographs; 312 provided minimum 2-year radiographs. Radiographic evaluation of both cohorts was done by an independent core laboratory (Medical Metrics) per the Knee Society recommendations^24^ by a fellowship-trained musculoskeletal radiologist using a detailed radiographic analysis protocol. The bone-cement and the implant-cement interfaces were evaluated across zones consistent with Knee Society recommendations^24^ based on implant geometry. Based on the timing of observation, an index radiolucent line (RLL) was one that was observed on immediate postoperative radiographs and would be most likely the result of surgical process challenges, such as poor cement interdigitation into the sclerotic bone^25,26^ because bone resorption would not be expected immediately after surgery. In this study, index RLLs were defined as interface gaps and could be later classified as a RLL if the width increased at subsequent intervals. By zone, the width of each RLL was recorded in millimeters and progression over time was assessed. RLLs were tallied by subject according to width: 0 to <1 mm, ≥1 mm to < 2 mm, and ≥ 2 mm. If a study subject had a RLL in more than one zone, the knee was only counted once, for the widest RLL. Final data for this study were collected in August 2018.

### Analysis Methodology

Statistical summaries and analyses were conducted with all available data at respective time points for all enrolled subjects. Data imputation methods were not used in cases of missing data. PROMs, AKS, and ROM comparisons were conducted with a 2-sided independent samples *t*-test. Because of multiple comparisons of these many continuous outcomes, a *P* value threshold of 0.01 for statistical significance was used for identifying differences that favored New-TKA. The large sample sizes in this study (eg, N > 600 subjects for Existing-TKA and N > 900 subjects for New-TKA at 2 years) were sensitive to small effects; an effect size of 0.18 would have been detected with a 2-sided alpha of 0.01 and 80% power. The Fisher exact test was used to compare complication rates, responder rates, and the percentage of subjects with radiographic findings. Implant survivorship was estimated with Kaplan-Meier (KM) methodology and compared across cohorts with a log-rank *P* value, where a revision was defined as the removal of any TKA implant for any reason (KM1), and the implant was considered to be surviving if it had not been revised. For subjects who were revised, the time to revision was the date of revision minus the date of primary TKA. The time to censoring for subjects who were not revised was defined to be the time of the last clinical study visit, death, or study withdrawal minus the date of primary TKA. KM methodology was also used to evaluate and compare implant survivorship with three other definitions of revision: the removal of any implant for any reason other than infection (KM2), the removal of metal implants for any reason (KM3), and the removal of any metal implants for any reason other than infection (KM4).

## Results

### Demographics and Follow-up Summaries

Demographics and follow-up summaries are presented in Table 1. Demographics were similar across cohorts and representative of a typical primary TKA population. The mean duration of follow-up was slightly longer for New-TKA because many of the subjects were at study centers who agreed to extend their follow-up by rolling over subjects into an ongoing 15-year study (NCT01754363).

**Table 1:** Demographics and Follow-up

Variable	Existing-TKA N = 752	New-TKA N = 1129
Age: mean (SD) (range)	65.7 (8.16) (28-80)	65.2 (7.73) (34-85)
Sex: n (%) female	439 (58.4)	659 (58.4)
BMI (kg/m^2^): mean (SD)	31.9 (6.35)	31.7 (5.79)
Primary diagnosis: n (%) OA	737 (98.0%)	1123 (99.5%)
Configuration: n (%)		
CR FB	186 (24.7%)	300 (26.6%)
CR RP	202 (26.9%)	243 (21.5%)
PS FB	154 (20.5%)	319 (28.3%)
PS RP	210 (27.9%)	267 (23.6%)
Sample size for PROMs and radiographs^a^: n PROMs; n radiographs		
Preoperative	747; 497	1127; 1118
1 year	664; 407	973; 950
2 years	636; 312	931; 922
Duration of follow-up^b^ (y): mean (SD)	2.2 (0.70)	2.5 (0.85)

CR = cruciate retaining, FB = fixed bearing, OA = osteoarthritis, PROMs = patient-reported outcome measures, PS = posterior-stabilized, RP = rotating platform, TKA = total knee arthroplasty

a n for PROMs reflects subjects with Knee Injury and Osteoarthritis Outcome Score-ADL; n for other PROMs varies; n for radiographs reflects subjects with radiographs on file; each view or assessable may vary.

b Duration of the follow-up was the time from index TKA to last PROMs or clinical follow-up or revision.

### Comparison of Patient-Reported Outcomes

Preoperatively, subjects reported similar functional status across all PROMs, AKS, and ROM. Point estimates of mean outcomes for PROMs, AKS, and ROM were all equal or better for New-TKA versus Existing-TKA at both 1 and 2 years, with many *P* values less than 0.01 (Table 2). Similarly, point estimates for nearly all changes from baseline means were better for New-TKA versus Existing-TKA at both 1 and 2 years; all that had a *P*-value < 0.01 favored New-TKA. The comparison of 2-year OMERACT-OARSI responder rates (moderate or high combined) and high responder rates favored New-TKA with *P* values < 0.05 (Table 3).

**Table 2:** PROMS, AKS, and ROM Outcomes, Including Change From Preoperative Baseline (CFB)

Outcome	Scale	Preoperative			1 Year			2 Years		
		Existing-TKA	New-TKA	*P*-Value^a^	Existing-TKA	New-TKA	*P*-Value^a^	Existing-TKA	New-TKA	*P*-Value^a^
		Mean (SD)	Mean (SD)		Mean (SD) CFB Mean (SD)	Mean (SD) CFB Mean (SD)		Mean (SD) CFB Mean (SD)	Mean (SD) CFB Mean (SD)	
KOOS										
Activities of daily living	0-100	49.2 (18.64)	49.0 (17.88)	0.8421	84.8 (15.61)	87.7 (14.23)	**0.0001**	86.8 (15.23)	89.0 (14.58)	**0.0051**
					*34.9 (19.87)*	*38.1 (19.32)*	***0.0012***	*36.8 (20.49)*	*39.3 (19.56)*	*0.0169*
Pain	0-100	45.7 (16.81)	44.0 (16.37)	0.0289	84.4 (16.61)	86.8 (15.19)	**0.0030**	87.1 (15.80)	88.9 (15.05)	0.0186
					*38.2 (20.36)*	*42.3 (19.79*)	***<0.0001***	*40.6 (20.59)*	*44.4 (19.70)*	***0.0002***
Symptoms	0-100	48.1 (18.91)	46.5 (18.23)	0.0617	78.4 (16.75)	80.5 (15.34)	**0.0092**	82.2 (15.35)	84.1 (14.80)	0.0168
					*30.2 (22.24)*	*33.4 (21.76)*	***0.0035***	*33.6 (21.95)*	*37.3 (21.34)*	***0.0011***
Sport and recreation	0-100	18.1 (20.41)	17.2 (19.63)	0.3911	53.7 (30.52)	59.7 (28.85)	**<0.0001**	58.8 (29.87)	63.9 (29.08)	**0.0010**
					*35.1 (31.22)*	*41.6 (29.96)*	***<0.0001***	*40.8 (31.79*)	*46.2 (29.75)*	***0.0008***
Quality of life	0-100	24.2 (17.62)	23.9 (17.35)	0.7247	69.5 (23.01)	73.2 (22.22)	**0.0010**	73.5 (23.00)	77.0 (22.54)	**0.0032**
					*44.6 (25.83)*	*48.8 (25.80)*	***0.0014***	*48.7 (26.64)*	*52.4 (25.19)*	***0.0045***
OKS	0-48	22.7 (8.07)	22.5 (7.88)	0.6200	40.1 (7.31)	41.3 (6.65)	**0.0011**	41.1 (6.82)	41.9 (6.86)	0.0273
					*17.0 (8.78)*	*18.4 (8.67)*	***0.0021***	*18.0 (8.94)*	*19.0 (8.71)*	*0.0241*
PKIP										
Overall	0-100	27.1 (14.90)	28.5 (13.26)	0.0477	71.2 (18.82)	73.9 (18.43)	**0.0033**	73.5 (19.37)	76.5 (19.24)	**0.0030**
					*43.8 (21.74)*	*45.1 (21.56)*	*0.2225*	*46.1 (22.59)*	47.3 (21.59)	*0.3263*
Confidence	0-10	3.6 (2.02)	3.7 (1.94)	0.1938	7.9 (1.89)	8.2 (1.84)	**0.0014**	8.1 (1.94)	8.4 (1.90)	**0.0037**
					*4.3 (2.44)*	*4.4 (2.35)*	*0.2060*	*4.4 (2.49)*	*4.5 (2.38)*	*0.5460*
Stability	0-10	3.4 (2.10)	3.4 (2.02)	0.4158	8.2 (1.96)	8.5 (1.88)	**0.0092**	8.3 (2.01)	8.6 (1.94)	**0.0061**
					*4.8 (2.46)*	*5.0 (2.42)*	*0.1708*	*4.9 (2.48)*	*5.0 (2.40)*	*0.3048*
Satisfaction	0-10	2.1 (1.64)	2.1 (1.55)	0.3166	7.9 (2.05)	8.1 (2.07)	0.0374	8.1 (1.98)	8.3 (2.05)	0.0415
					*5.8 (2.50)*	*6.0 (2.51)*	*0.3140*	*6.0 (2.49)*	*6.1 (2.47)*	*0.3826*
Modifying activities	0-10	3.6 (2.83)	4.0 (2.85)	0.0162	6.2 (3.31)	6.4 (3.42)	0.4370	6.4 (3.33)	6.6 (3.46)	0.3336
					*2.6 (4.30)*	*2.4 (4.45)*	*0.4604*	*2.8 (4.22)*	*2.6 (4.57)*	*0.4544*
EQ 5D	−1 to 1	0.64 (0.193)	0.63 (0.187)	0.6250	0.87 (0.147)	0.88 (0.137)	0.0646	0.88 (0.139)	0.88 (0.153)	0.7366
					*0.22 (0.208)*	*0.24 (0.195)*	*0.1247*	*0.24 (0.210)*	*0.23 (0.203)*	*0.8765*
Original AKS^b^	0-100	39.1 (14.94)	38.8 (15.15)	0.7487	87.6 (13.12)	88.4 (12.66)	0.2776	88.0 (11.97)	89.6 (11.98)	0.0458
					*47.8 (18.15)*	*49.4 (18.35)*	*0.1705*	*48.5 (17.77)*	*50.2 (18.59)*	*0.1683*
ROM^b^	0-180	108.7 (15.94)	107.8 (15.94)	0.3007	118.5 (10.06)	118.9 (12.27)	0.5250	119.0 (11.12)	119.6 (12.27)	0.4473
					*7.8 (14.76)*	*10.4 (15.67)*	***0.0050***	*8.0 (14.70)*	*11.7 (16.98)*	***0.0005***

AKS = American Knee Society Score, CFB = change from baseline, KOOS = Knee Injury and Osteoarthritis Outcome Score, OKS = Oxford Knee Score, PROM = patient-reported outcome measure, PKIP = Psychometric Evaluation of the Patient's Knee Implant Performance, ROM = range of motion (flexion minus extension), TKA = total knee arthroplasty

a *P* values < 0.01 are shown in bold.

b The Original AKS and ROM are not considered PROMs but are included in this table. The 2011 AKS was collected for New-TKA, along with the original AKS pain score to allow for original AKS scoring of New-TKA. Core-laboratory radiographic assessment of tibiofemoral alignment was used for both Existing-TKA and New-TKA.

Numbers in italics denote CFB summaries.

**Table 3:** OMERACT-OARSI Responder Analysis at 2 years

Criteria	Existing-TKA^a^	New-TKA^a^	*P*-Value
WOMAC			
High responder	76.3% (476/624)	81.2% (736/906)	0.0210
Moderate or high responder	90.6% (572/631)	93.9% (866/922)	0.0177
KOOS			
High responder	76.7% (487/635)	82.8% (770/930)	0.0035
Moderate or high responder	91.5% (579/633)	94.2% (877/931)	0.0420

KOOS = Knee Injury and Osteoarthritis Outcome Score, OMERACT-OARSI = Osteoarthritis Research Society International—Outcome Measures in Rheumatology, TKA = total knee arthroplasty, WOMAC = Western Ontario and McMaster Universities Osteoarthritis Index

a Sample sizes vary by criteria because of subjects with missing end points (WOMAC or KOOS subscores).

### Comparison of Kaplan-Meier Implant Survivorship

A total of 11 Existing-TKA and 20 New-TKA subjects underwent revision of any implant for any reason, and the KM implant survivorship results for this definition (KM1) are presented in Table 4; the log-rank *P* value comparing survivorship was 0.9994. The curves in Table 4 are illustrated until only 40 subjects remain with additional follow-up. Three of the New-TKA revisions occurred after 3 years. The reason and timing of each revision are provided in Table 5, which shows that six Existing-TKA and eight New-TKA involved the removal of metal TKA implants (tibial or femoral). KM implant survivorship analyses for definitions KM2, KM3, and KM4 were conducted, yielding log-rank *P* values of 0.7251, 0.4881, and 0.4954, respectively. The Existing-TKA versus New-TKA 3-year KM implant survivorship estimates with definition KM2 were 98.6% versus 99.0%; with definition KM3, these were 99.1% versus 99.2%; and with definition KM4, these were 99.4% versus 99.7%. These analyses demonstrated that there was no notable difference in KM implant survivorship between cohorts.

**Table 4:** KM Implant Survivorship Estimates (KM1)

	1-year KM Survivorship (95% CI) N With Later Follow-up (Cumulative Revised)	2-year KM Survivorship (95% CI) N With Later Follow-up (Cumulative Revised)	3-year KM Survivorship (95% CI) N With Later Follow-up (Cumulative Revised)
Existing-TKA (N = 752)	99.7% (98.9, 99.9) N = 706 (2 Revised)	98.5% (97.3, 99.2) N = 548 (10 Revised)	98.3% (97.0, 99.1) N = 70 (11 Revised)
New-TKA (N = 1129)	99.3% (98.6, 99.6) N = 1099 (8 Revised)	98.7% (97.9, 99.2) N = 924 (14 Revised)	98.1% (96.8, 98.9) N = 258 (17 Revised)

CI = confidence interval, KM = Kaplan-Meier, KM1 = Kaplan-Meier survivorship for removal of any TKA implant for any reason, TKA = total knee arthroplasty

**Table 5:** Reasons for Revision, Timing, and Implants Removed (Highlighted Cells Indicate Removal of Metal Implants)

Existing-TKA (N = 752) 11 Revisions			New-TKA (N = 1129) 20 Revisions		
Revision Reason	Time (yr) at Revision	Implants Removed^a^	Revision Reason	Time (yr) at Revision	Implants Removed^a^
Infection	0.5	F, T, I, P	Bone fracture	0.3	F, T, I, P
	1.1	I		1.2	P
	1.5	F, T, I, P	Crepitus	0.9	I
	2.0	I	Infection	0.0	I
Instability	1.2	F, I		0.6	P
Loosening	1.7	F, T, I, P		1.7	F, T, I, P
Pain	0.6	I		2.4	F, T, I, P
	1.0	F, T, I		2.4	F, T, I
	1.7	P		2.6	I
	1.8	I	Loosening	1.9	F, T, I
	1.8	F, T, I, P	Pain	1.6	I
				1.8	I
				3.5	F, T, I
			Stiffness	0.4	I
				0.4	I
				0.4	F, T, I
				0.9	I
				1.2	I
				3.2	F, I
				3.9	I

TKA = total knee arthroplasty

a F=femoral, T=tibial, I=insert, P=patella

Table 6 presents a comparison of the number and percentage of Existing-TKA versus New-TKA subjects who experienced local AEs. Aside from pain, which was higher for New-TKA (*P* = 0.0235), and symptomatic crepitus, which was higher for Existing-TKA (*P* = 0.0057), the distribution of local AEs was similar between cohorts.

**Table 6:** Comparison of Subjects With Local AEs

AE^a^	Existing-TKA (N = 752)		New-TKA (N = 1129)		Fisher Exact *P*
	No. of Subjects^b^	% of Subjects	No. of Subjects^b^	% of Subjects	
Stiffness	75	10.0	85	7.5	0.0640
Pain	21	2.8	56	5.0	0.0235
Infection	18	2.4	27	2.4	1.0000
Effusion	11	1.5	30	2.7	0.1060
Crepitus-asymptomatic	15	2.0	13	1.2	0.1732
Instability	12	1.6	14	1.2	0.5490
Crepitus-symptomatic	15	2.0	6	0.5	0.0057
Trauma	5	0.7	15	1.3	0.2506
Wound complication	8	1.1	7	0.6	0.3016
Bone fracture	4	0.5	13	1.2	0.2159
Tendinitis	6	0.8	6	0.5	0.5588
Neuralgia	2	0.3	8	0.7	0.3322
Loosening	3	0.4	1	0.1	0.3082

AE = adverse event, TKA = total knee arthroplasty

a The following each occurred in < 0.4% of the subjects: hemarthrosis, bursitis, patellar clunk, hematoma, inflammation, tendon rupture, DVT, femoral notching, osteonecrosis (patella), spin out, and vascular.

b Subjects were only counted once for a respective AE, even if they reported the AE more than once.

### Comparison of Radiographic Results

Radiographic outcomes are presented in Table 7. At the implant/cement interface, the first postoperative radiographs demonstrated similar rates of interface gaps. At the bone/cement interface, the immediate postoperative radiographs demonstrated statistically lower rates of interface gaps for all implants in the New-TKA. For the Existing-TKA cohort, interface gaps were observed at both the femoral and tibial bone/cement interfaces in 8% of the knees while for New-TKAs, they were observed near the femoral implant in 1.6% of the TKAs and near the tibial base in 3.4% of the TKAs. At later intervals, the width of most of the RLLs was in the ≥1 mm to <2 mm category with a very low incidence of RLL ≥2 mm in either cohort at the implant/cement or bone/cement interfaces. In addition, wider RLLs (≥2 mm) and those that were also progressive were rare in both groups.

**Table 7:** Radiographic Outcomes—Including Postoperative Interface Gaps and Radiolucencies at Implant/Cement and Bone/Cement Interfaces

Immediate Postoperative Finding		Existing-TKA^a^	New-TKA^a^	*P*
Implant/Cement interface gap	Femoral	6/341 (1.8%)	28/1056 (2.7%)	0.4236
	Tibial	9/341 (2.6%)	15/1056 (1.4%)	0.1499
Bone/Cement interface gap	Femoral	26/341 (7.6%)	17/1056 (1.6%)	< 0.0001
	Tibial	27/341 (7.9%)	36/1056 (3.4%)	0.0013
1-year finding				
Implant/Cement RLL ≥ 2 mm	Femoral	2/381 (0.5%)	1/936 (0.1%)	0.2025
	Tibial	0/392 (0%)	0/944 (0%)	NA
Implant/Cement RLL ≥ 2 mm and progressive	Femoral	0/381 (0%)	1/936 (0.1%)	1.00
	Tibial	0/392 (0%)	0/944 (0%)	NA
Bone/Cement RLL ≥ 2 mm	Femoral	6/381 (1.6%)	0/935 (0%)	0.0006
	Tibial	4/393 (1.0%)	1/943 (0.1%)	0.0284
Bone/Cement RLL ≥ 2 mm and progressive	Femoral	0/381 (0%)	0/935 (0%)	NA
	Tibial	0/393 (0%)	1/943 (0.1%)	1.00
2-year finding				
Implant/Cement RLL ≥ 2 mm	Femoral	1/292 (0.3%)	1/906 (0.1%)	0.4282
	Tibial	1/297 (0.3%)	1/914 (0.1%)	0.4305
Implant/Cement RLL ≥ 2 mm and progressive	Femoral	0/292 (0%)	1/906 (0.1%)	1.00
	Tibial	0/297 (0%)	0/914 (0%)	NA
Bone/Cement RLL ≥ 2 mm	Femoral	6/292 (2.1%)	0/906 (0%)	0.0002
	Tibial	2/297 (0.7%)	3/912 (0.3%)	0.6016
Bone/Cement RLL ≥ 2 mm and progressive	Femoral	2/292 (0.7%)	0/906 (0%)	0.0593
	Tibial	0/297 (0%)	3/912 (0.3%)	1.00

RLL = radiolucent line, TKA = total knee arthroplasty

a Different denominators reflect the number of assessments, which differ by interval and also by the number of assessable radiographs.

## Discussion

It is well documented that TKA can reduce pain and improve function, but a notable percentage of patients are incompletely satisfied. Newer implants are designed to improve these suboptimal outcomes. This study was designed to carefully document a wide array of outcomes, with a focus on PROMs, of one specific, newer TKA design using the same manufacturer's predecessor implant as a control. Using the same surgeons to implant both Existing-TKA and New-TKA was an effort to minimize outcome differences and bias due to differences in surgical technique, patient populations, pain management, etc.

Postoperatively, subjects in both cohorts reported statistically significant improvements in PROMs compared with preoperative baseline. At both 1 and 2 years postoperatively, PROMs showed small differences favoring New-TKA compared with Existing-TKA. These differences were modest in magnitude, and although statistically significant, they were less than any recognized minimum clinically important differences for these PROMs. This is expected because TKAs, in general, have been shown to have a positive effect on patients' quality of life.^27^ It can be difficult to determine whether these small differences in outcomes signify clinical significance, and for that reason, we performed the OMERACT-OARSI^22,23^ responder analysis to further enhance the interpretation of the results. Approximately 3% to 6% more of the New-TKA subjects were responders compared with Existing-TKAs, inclusive of both responder criteria. Whether this is clinically significant and remains to be seen, but in the short term, we can safely conclude that the new implant shows equivalent, if not somewhat better clinical outcomes. Health economic literature focused on cost-effectiveness modeling evidence, which shows that even small improvements in PROMs can markedly raise cost-effectiveness favorability when translated into gains in quality-adjusted life years, provided that the improvement is sustained over long periods of time.^28^

The 3-year KM implant survivorship rates (revision defined as the removal of any implant for any reason) demonstrated no statistically significant difference (log-rank, *P* = 0.9994) between cohorts and were similar to 3-year estimates for the New-TKA and the class of TKAs from national joint registries^2,29,30^ and similar to the 97.68% implant survivorship estimate from the Michigan Arthroplasty Registry^31^ and the recent Kaiser Registry presentation.^32^ The reasons for revisions in the two cohorts in this study were similar at this time point. There were slightly more revisions for stiffness in New-TKA versus Existing-TKA, and these seven cases were across six different study sites, with no apparent pattern. Furthermore, the aseptic loosening rate in both cohorts was low and similar, which complements two RSA studies that showed no difference in maximum total point motion versus SIGMA in one study^33^ and maximum total point motion consistent with published criteria in the other study.^34^ These study data provide a large data set on New-TKA with which to understand a robust, short-term device survivorship estimate.

This is the first study to review a sizable quantity of New-TKA radiographs and compare them with a clinically successful product. The results from the independent radiographic reviewer's assessment of the metal-cement and the bone-cement interfaces out to two years demonstrated that New-TKA has similar results compared with Existing-TKA. The type and frequency of local AEs were similar across cohorts. The rate of symptomatic crepitus for New-TKA was observed to be slightly lower than for Existing-TKA; this is consistent with the comparison of patellofemoral complications among posterior stabilized (posterior-stabilized fixed bearing and posterior-stabilized rotating platform) subjects from these same two studies that was reported separately.^35^

The strengths of this study include prospective data collection with fairly large sample sizes, consecutive enrollment, and multicenter data coming from experienced surgeons/clinical researchers who included all consecutive knees starting with their first New-TKA implanted. Additional strengths include the use of multiple validated PROMs to better understand the patient's perspective; utilization of knee-specific PROMs that included more advanced activities, such as KOOS Sports and Recreation, and a newer PROM (Patient's Knee Implant Performance) that included questions related to underlying reasons associated with functional outcomes, such as confidence and stability when performing activities. An independent radiographic core laboratory reviewed both cohorts of radiographs using an identical protocol, and 12 sites in the New-TKA cohort have elected to continue follow-up to 15 years thus providing opportunity to follow a portion of this original cohort long term. Approximately half of the subjects in both cohorts come from the United States, which currently does not have product-level reporting in their registry^36^; thus, this study provides a broader view of the available implant survivorship information for this new product beyond what is currently available in published registry reports.^1,2,29^

The weaknesses of this study included nonrandomized enrollment, which was balanced by the fact that most of the sites participated in both cohorts to minimize biases associated with institutional practices. A potential for temporal bias was observed because Existing-TKA enrollment preceded New-TKA enrollment at sites which participated in both studies; however, all implants were placed over a fairly similar time frame, so it is unlikely that any new protocols or surgical techniques influenced the outcomes. Incomplete radiographic review of Existing-TKA was observed, and implementation of the AKS 2011 required a conversion to compare with the previous AKS outcomes.

Although improvements out to 2 years that are demonstrated with the New-TKA in this study are subtle, we are encouraged that the new design has resulted in PROMs that are moving in a positive direction. With many examples of implant “improvements” in the orthopaedic industry leading to poorer performance, these data show that collaboration between clinicians and industry to improve outcomes in TKA can produce positive results. The early PROMs and radiographic outcomes of the New-TKA have produced encouraging results with modest improvement in most PROMs while maintaining the low level of radiolucency of the Existing-TKA, which suggests satisfactory long-term implant survival.

The authors recommend that new implants that are introduced undergo similar levels of scrutiny using multicenter surveillance evaluation as recommended by several landmark articles^11,37^ and including a variety of PROMs to carefully assess performance from a patient perspective and radiographic/adverse event data to characterize the safety profiles. A longer term follow-up is ongoing for many of the study sites who have chosen to join the noted ongoing 15-year New-TKA study.
